# Syndrome de Meckel Gruber: à propos d’un cas rare

**DOI:** 10.11604/pamj.2016.25.43.9696

**Published:** 2016-09-29

**Authors:** Sanaa Itchimouh, Karima Khabtou, Sakher Mahdaoui, Houssine Boufettal, Naima Samouh

**Affiliations:** 1 Service de Gynécologie Obstétrique ‘C’, CHU Ibn Rochd, Casablanca, Maroc

**Keywords:** Syndrome de Meckel, dysplasie rénale, encéphalocèle, diagnostic anténatal, Meckel syndrome, renal dysplasia, encephalocele, prenatal diagnosis

## Abstract

Le syndrome de Meckel Gruber est un syndrome poly malformatif rare, de transmission autosomique récessive, défini par d'encéphalocèle occipital, polydactylie et dysplasie kystique rénale. L'échographie constitue, à l'heure actuelle, le meilleur moyen de dépistage anténatal de cette poly malformation létale et sa confirmation se fait par l'étude du caryotype. Nous rapportons un cas de syndrome de Meckel découvert par échographie. La grossesse a été interrompue à 25 semaines d'aménorrhée.

## Abstract (en)

Meckel Gruber syndrome is a rare autosomal recessive polymalformation syndrome characterized by occipital encephalocele, polydactyly and cystic renal dysplasia. Ultrasound is, at present, the best tool for prenatal screening of this lethal polymalformation and diagnosys is confirmed by karyotyping. We here report a case of Meckel Gruber syndrome detected by ultrasound. Abortion was performed at 25 weeks of amenorrhoea.

## Introduction

Le syndrome de Meckel Gruber décrit par Meckel en 1822, et Gruber en 1934 associant une encéphalocèle, dysplasie kystique des reins, et polydactylie. La variabilité des tableaux cliniques recensés dans la littérature montre que le polymorphisme de ce syndrome est une caractéristique essentielle. L´échographie constitue, à l´heure actuelle, le meilleur moyen de dépistage anténatal de cette poly malformation létale [1]. Nous rapportons un cas de syndrome de Meckel découvert à l'échographie sur grossesse de 26 SA+2 jours.

## Patient et observation

**Figure 1:**
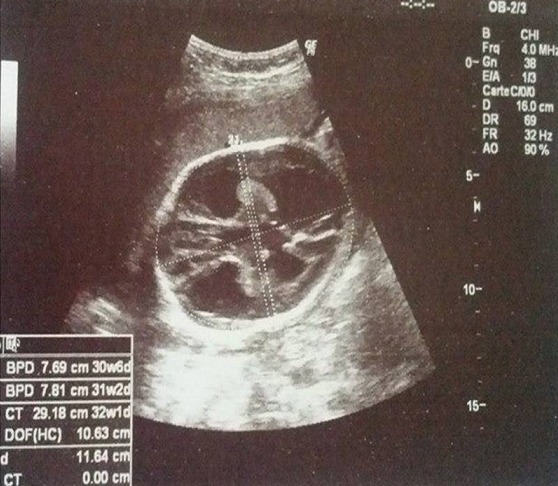
Aspect échographique d’une hydrocéphalie majeure

**Figure 2:**
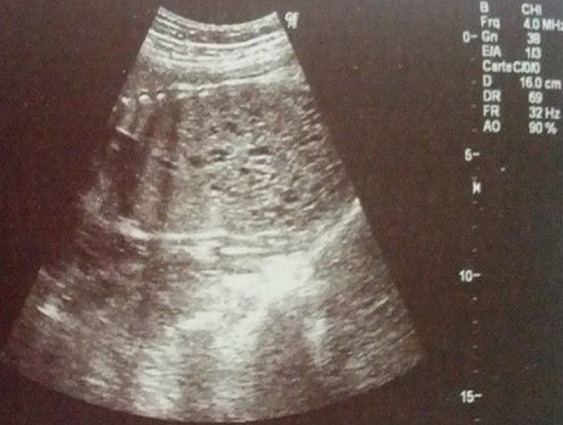
Aspect échographique de kystes rénaux

**Figure 3:**
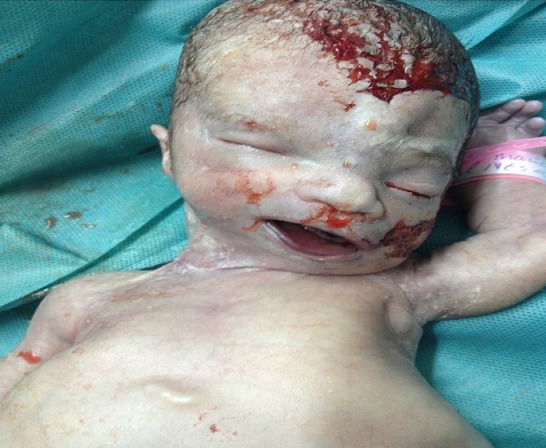
Aspect de rétrognatisme neonatal

**Figure 4:**
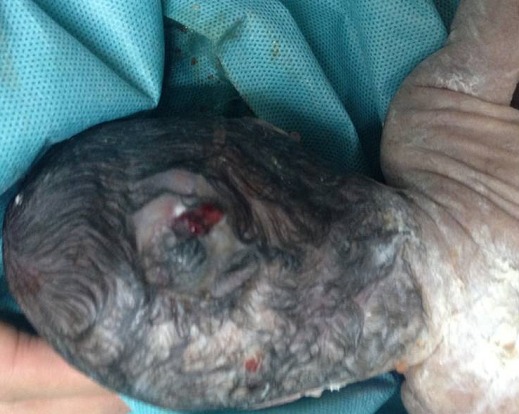
Encéphalocèle postérieur

**Figure 5:**
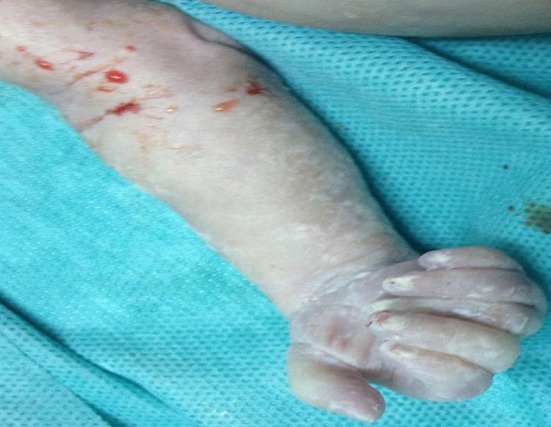
Polydactylie sur les 4 segments distaux

**Figure 6:**
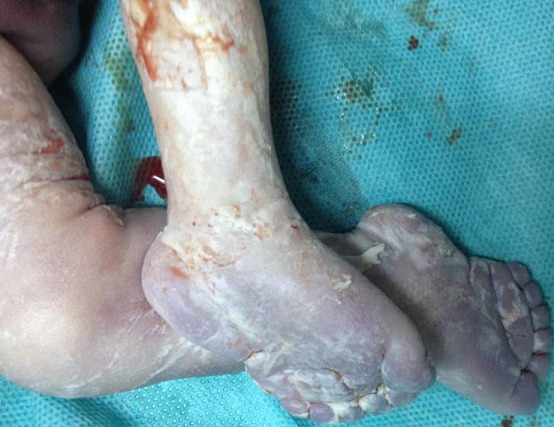
Aspect de pieds bots avec polydactylie

**Figure 7:**
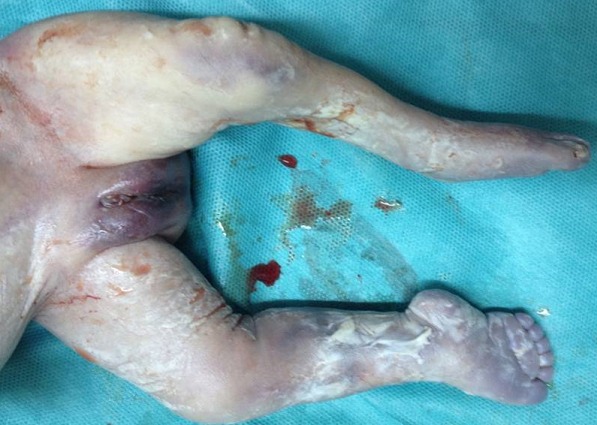
Aspect incurvé de l’humérus avec aspect normal des organes génitaux externes

Patiente âgée de 24 ans, primigeste primipare, ayant comme antécédent une consanguinité de premier degré. Elle avait consulté pour la première fois pour suivi de grossesse de 26SA + 2 jrs. Une échographie morphologique fœtale a été réalisée objectivant une grossesse monofoetale évolutive avec malformations fœtales comprenant ; hydrocéphalie majeure (Figure 1) + syndrome poly malformatif rénal (Figure 2) et cardiaque associé à un anamnios sévère. L'interruption médicale de grossesse a été proposée et acceptée par le couple, elle a abouti à un accouchement par voie basse d'un fœtus de sexe féminin, poids 1100g. L'examen macroscopique retrouve: au niveau du pole céphalique: rétrognatisme (Figure 3) et encéphalocèle postérieur (Figure 4); au niveau de l'abdomen: une hépato splénomégalie avec ascite; au niveau des membres : une polydactylie (Figure 5) sur les 4 segments distaux, pieds bots (Figure 6) et un aspect incurvé de l'humérus (Figure 7); examen du rachis et les organes génitaux externes est normal. L'examen autopsique a été refusé par la famille. Devant ce syndrome poly malformatif, le diagnostic de syndrome de Meckel a été évoqué.

## Discussion

### Définition

Le syndrome de Meckel est un syndrome poly malformatif congénital rare, de transmission autosomique récessive, décrit pour la première fois en 1822 dans la littérature allemande. Le syndrome de Meckel est un syndrome héréditaire caractérisé par un ensemble de malformations congénitales touchant notamment le syndrome nerveux central et les reins. Il est généralement fatal peu après la naissance [2].

### Epidémiologie

Le syndrome de Meckel touche 1 personne sur 13 250 à 1 140 000 personnes dans le monde. Plus fréquent en Finlande, où sa prévalence à la naissance est de 1 pour 9 000 et où la fréquence de la mutation est de 1% [3]. Trois gènes ont été cartographiés: MKS1 sur le chromosome 17, MKS2 sur le 11, et MKS3 sur le 8.

### Manifestation clinique

Le syndrome de Meckel est une maladie monogénique caractérisée par la combinaison de kystes rénaux et d'autres manifestations [4]: anomalies de développement du système nerveux central (encéphalocèle occipitale); polydactylie; dysplasie des voies biliaires et des kystes hépatiques. Le syndrome de Meckel est défini généralement par la triade: Encéphalocèle occipital, dysplasie kystique des reins et polydactylie. La polydactylie est le plus souvent post-axiale (6ème doigt), mais peut parfois être pré-axiale (duplication du pouce) Une incurvation des os longs des membres est présente dans un cas sur 6. D´autres anomalies peuvent être présentes: fente labio-palatine, anophtalmie ou une microphtalmie, atrésie de l´urètre, malformations cardiaques et des organes génitaux.

### Critères diagnostiques

critères majeurs: la dysplasie rénale kystique est un critère obligatoire pour établir le diagnostic associé à un anamnios [5–7]; critères mineurs: la fibrose hépatique; l'encéphalocèle occipitale; polydactylie; autres malformations du système nerveux central: malformation de Dandy-Walker et malformation d´Arnold Chiari.

### Diagnostic anténatal

Le diagnostic prénatal peut être réalisé à partir d´une image échographique de kyste anéchogène intracrânien et/ou d´une malformation crânienne à la fin du premier trimestre ou encore en présence de reins anormalement gros [8]. Les autres caractéristiques du syndrome peuvent être décelées plus tardivement à l´échographie. L´amniocentèse peut révéler un taux élevé d´alpha-foetoprotéine amniotique dû à l'encéphalocèle [9]. Le caryotype reste le meilleur moyen pour confirmer le diagnostic Si la grossesse est menée à terme, le nouveau-né décède en période périnatale.

### Evolution

Le syndrome de Meckel est létal avec en moyenne une survie inférieure à 24 heures. Toutefois Genuardi décrit un cas de syndrome de Meckel comprenant au départ des reins polykystiques, un Dandy-Walker, une polydactylie postaxiale et ayant survécu 43 mois avant de décéder par une insuffisance rénale [10]. Le conseil génétique a pour objectif d´informer les parents d´un individu atteint que le risque de récurrence est de 25% pour les grossesses ultérieures.

### Diagnostic différentiel

Les trisomies 13 et 18 sont éliminées devant un caryotype normal [11]. D´autres syndromes poly malformatifs peuvent poser des difficultés diagnostiques plus importantes. Le syndrome de Carpenter-Hunter associe encéphalocèle, dysplasie kystique rénale, polydactylie, mais aussi des lésions osseuses généralisées. On retrouvera aussi la polydactylie dans les syndromes d'Ellis von Creveld, polydactylie côtes courtes, Moon-Bardet-Biedl, holoprosencéphalie-polydactylie (pseudo-trisomie 13). Une grande aide au diagnostic sera apportée par l´isolement du gène du syndrome de Meckel.

### Conseil génétique

Le syndrome de Meckel est de transmission autosomique récessive. La fréquence du gène du syndrome de Meckel dans la population générale est de l´ordre de 1/400. A l´heure actuelle, il est encore impossible de dépister précisément le gène du syndrome de Meckel. Toutefois la localisation récente du locus responsable au niveau du chromosome 17 avec trois gènes potentiellement impliqués montre que la recherche est proche du but. C´est pourquoi, en l´absence de critères formellement définis pour le syndrome de Meckel, il est indispensable de conserver des tissus fœtaux pour une analyse génique et moléculaire qui permettra un diagnostic précis. En effet, si dans le cas d´un Dandy Walker isolé le conseil génétique doit être rassurant, le risque de récidive locale étant de 1%, en cas de syndrome de Meckel avec Dandy Walker ce risque est de 25% [12].

## Conclusion

Les malformations les plus fréquemment retrouvées dans le syndrome de Meckel sont la dysplasie rénale polykystique, l'encéphalocèle, la polydactylie, et la fibrose hépatique, aucune d'entre elles n'est constante. Le polymorphisme de ce syndrome peut être considéré comme une caractéristique essentielle, mais qui complique l'accès à un diagnostic de certitude. Les progrès de la génétique avec l'isolement précis du gène responsable du syndrome de Meckel vont constituer l'étape ultérieure vers un diagnostic de certitude, et son application au diagnostic anténatal.
